# Updating the Aesthetic Fluency Scale: Revised long and short forms for research in the psychology of the arts

**DOI:** 10.1371/journal.pone.0281547

**Published:** 2023-02-08

**Authors:** Katherine N. Cotter, Rebekah M. Rodriguez-Boerwinkle, Alexander P. Christensen, Anna Fekete, Jeffrey K. Smith, Lisa F. Smith, Pablo P. L. Tinio, Paul J. Silvia

**Affiliations:** 1 Positive Psychology Center, University of Pennsylvania, Philadelphia, Pennsylvania, United States of America; 2 Department of Psychology, University of North Carolina at Greensboro, Greensboro, North Carolina, United States of America; 3 Department of Psychology and Human Development, Peabody College, Vanderbilt University, Nashville, Tennessee, United States of America; 4 Department of Cognition, Emotions and Methods in Psychology, University of Vienna, Vienna, Austria; 5 College of Education, University of Otago, Dunedin, Otago, New Zealand; 6 Educational Foundations Department, Montclair State University, Montclair, New Jersey, United States of America; Aalborg University, DENMARK

## Abstract

People’s knowledge about the arts shapes how they experience and engage with art. Since its introduction, the 10-item Aesthetic Fluency Scale has been widely used to measure self-reported art knowledge. Drawing from findings and researchers’ experience since then, the present work develops and evaluates a Revised Aesthetic Fluency Scale using item response theory to broaden its scope (36 items) and refine its response scale. In a large sample (*n* = 2,089 English-speaking adults), Study 1 found strong evidence for unidimensionality, good item fit, and a difficulty level suitable for its targeted population; Study 2 (*n* = 392) provided initial evidence for score validity via relationships with art engagement, Openness to Experience, and aesthetic responsiveness; and Study 3 derived a brief, 10-item form for time-constrained projects. Taken together, the revised scales build upon lessons learned from the original and appear promising for the next generation of research.

## Introduction

Art knowledge is one of the most fundamental variables in the psychology of aesthetics and the arts. The beliefs, knowledge, and expertise that people bring to art encounters are central to developmental [1] and cognitive [2–4] theories of how people view, think about, and experience the arts. Beyond basic research, understanding how people gain and use art knowledge is central to the applied and translational goals of advancing art education and the public appreciation of the arts [5].

In the present research, we develop a revised version of the Aesthetic Fluency Scale [6], one of the most widely used self-report tools for measuring individual differences in art knowledge. After reviewing past work with the scale, we identify key areas for improvement and describe the iterative development of a revised scale with psychometric tools from classical test theory, item response theory, and network models. We then present evidence for score validity and conclude with a brief form of the scale for use when time and survey space are tight.

### The original Aesthetic Fluency Scale

Researchers interested in measuring art knowledge have used a few measurement strategies, each with different strengths and drawbacks. One approach is to measure demographic markers of art expertise, such as whether people have earned degrees, pursued formal training, or been employed in some domain of the arts [7, 8]. Another approach is to test people’s knowledge on questions about art that have correct answers. The VAIAK, for example, contains a section that assesses objective art knowledge [9, 10]. Finally, a third approach is to measure what Specker et al. [9] call *subjective knowledge*—people’s self-reported knowledge of the arts.

The Aesthetic Fluency Scale, developed by Smith and Smith [6], is a typical example of the subjective knowledge approach. According to the authors, “Aesthetic fluency is the knowledge base concerning art that facilitates aesthetic experience in individuals. It can be acquired through direct instruction, but it can also be learned through experience” (p. 50). The original 10-item Aesthetic Fluency Scale was developed during the early 2000s with visitors to the Metropolitan Museum of Art in New York. The respondents indicated how much they knew about 10 art ideas and artists: *Mary Cassatt*, *Isamu Noguchi*, *John Singer Sargent*, *Alessandro Botticelli*, *Gian Lorenzo Bernini*, *Fauvism*, *Egyptian Funerary Stelae*, *Impressionism*, *Chinese Scrolls*, and *Abstract Expressionism*. Responses were given using a 5-point scale:

0: I have never heard of this artist or term
1: I have heard of this but don’t really know anything about it
2: I have a vague idea of what this is
3: I understand this artist or idea when it is discussed
4: I can talk intelligently about this artist or idea in art

In a sample of 400 visitors, the scale showed good psychometric properties regarding internal consistency and unidimensionality [6].

Since then, the Aesthetic Fluency Scale has been widely used in research to measure variation in art knowledge [11–18]. Beyond its original fine arts form, a few variations on the method have appeared, such as a version for film knowledge [19], as have translations of the scale into other languages [11]. The core assessment approach—present participants with art-related figures and terms and record their self-reported knowledge—has inspired similar scales, such as the Art Affinity Index [20], that contain subjective art knowledge items. Taken together, the Aesthetic Fluency Scale has been both popular and fertile in arts research.

### Some directions for revision

Because the Aesthetic Fluency Scale has established itself as a prominent tool in arts research, it is worth taking stock of the scale to see how it might be refined and improved for the next generation of research. Based on the accumulated literature with the scale and on our experience as researchers who use it, we can see a few fruitful directions for revision.

First, it would be useful to have both long and short versions of the Aesthetic Fluency Scale. The original 10-item scale was grounded in field research in museum settings, where participants’ time is limited and concise scales are optimal. A crisp, 10-item scale is well-suited to such contexts, where researchers need brevity over breadth. Since the scale’s development, however, investigators in basic research settings, such as lab and survey research, have emerged as the largest group of scale users. For settings in which survey length is less of a constraint, a longer scale with a broader range of item content would be useful. Such a scale would likely yield more reliable scores and capture a wider universe of art-related concepts.

Second, the original Aesthetic Fluency Scale is probably too hard for how it is used. The original scale grew out of research in museum settings, particularly the Metropolitan Museum of Art. Such “bucket list” museums undoubtably attract a diverse audience, but the audience as a whole probably has more art interest and knowledge than the kinds of samples recruited for basic research. Many researchers use the scale for samples that are predominantly art novices, such as young adults enrolled in college courses or unselected samples from online panels. The original scale, which was developed with a relatively more expert sample than might typically be encountered in other research settings, might be less apt for less knowledgeable samples. A recent item response theory (IRT) analysis of the scale found that the scale as a whole was very hard for art novices—the test information function peaked at a high trait level—so the scale does not differentiate between people with lower levels of knowledge especially well [21]. This kind of “mistargeting” is common when researchers use a scale designed for one population with a population possessing a different average ability level.

Finally, the response scale could be simplified in two ways: streamlining the wording of each option and reducing the number of response options. Simplifying the wording and reducing the reading level of each response option should improve comprehension and readability, especially on mobile devices like tablets and smartphones that are increasingly used to take surveys. Furthermore, slimming down the original 5-point scale to a 3-point scale may make each response category more meaningful and distinct to participants. A theme in the Rasch and IRT literatures is that self-report scales commonly present respondents with too many response options [22–24]. Just because researchers offer a 7-point scale, for example, does not mean that respondents can reliably distinguish between seven levels of what is being assessed. In many cases, offering fewer options yields more orderly and reliable uses of the response scale.

**Fig 1:**
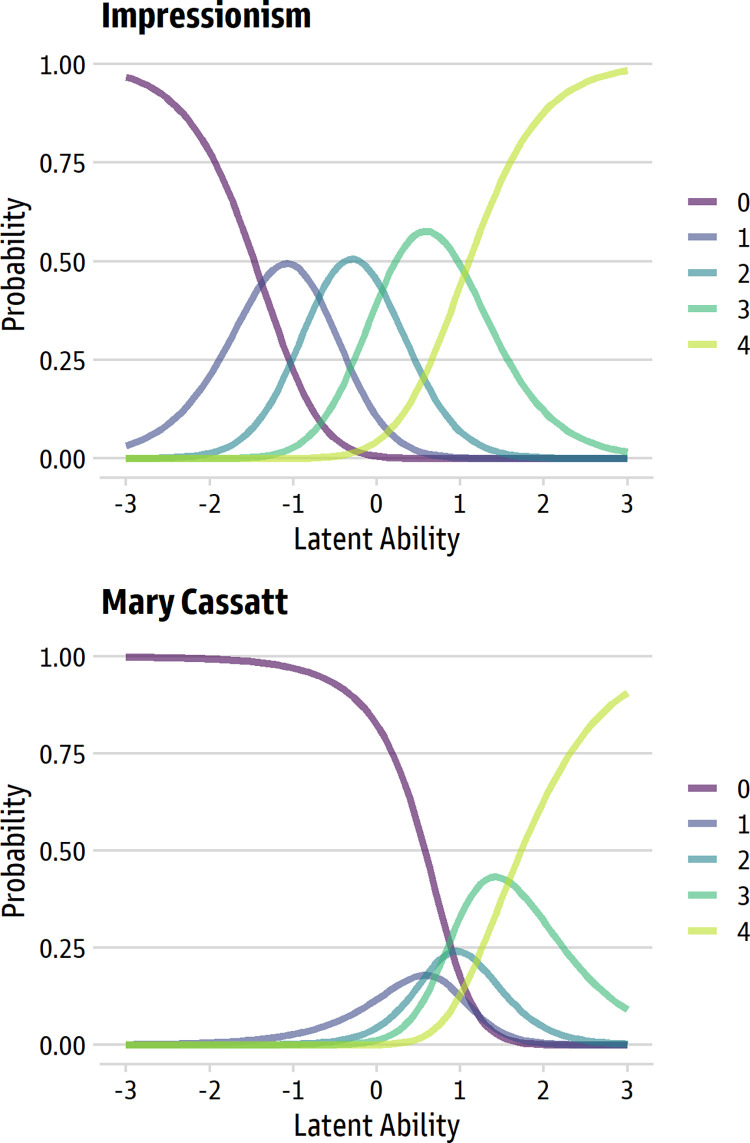
Examples of ordered and disordered thresholds for two items from the original Aesthetic Fluency Scale. The top panel (“Impressionism”) shows ordered category thresholds; the bottom panel (“Mary Cassatt”) shows disordered thresholds typical of items for the original scale.

As an example, Fig 1 displays category probability curves for two items from the original 10-item Aesthetic Fluency Scale. The responses are from a generalized partial credit model applied to the sample of more than 3200 people who were analyzed in the prior IRT analysis [21]. In the top panel, we see an ideal set of ordered response curves for the *Impressionism* item: as the trait level increases, the most likely response option moves from the lowest (0) to the highest (4) in ordered steps. Each of the five response options is, at some point, the most likely response, so each option in the five-point scale uniquely maps onto some region of the underlying trait.

The bottom panel, in contrast, uses the *Mary Cassatt* item to illustrate the more common kind of item response pattern in the original Aesthetic Fluency Scale. For this item, the response scale is disordered: as the trait moves from low to high, the most likely response on the 0–4 scale moves from 0 to 3 to 4. For two of the response options (1 and 2), there is no region of the underlying trait where the response is the most probable one. Such patterns in large, diverse samples usually indicate that there are some response options that are rarely endorsed, so a smaller response scale might be more suitable for how the intended respondents use the response scale [24].

### The present research

In the present research, we sought to revise the Aesthetic Fluency Scale in light of lessons learned from the past 15 years of its use. In Study 1, with a large, international sample (*n* = 2089), we outline the development of the 36-item Revised Aesthetic Fluency Scale, describe its psychometric features based on classical and IRT methods, and consider preliminary evidence for score validity. In Study 2, we examine additional evidence for score validity by relating the scale to well-known predictors of art knowledge, interest, and engagement. Finally, in Study 3, we describe the development of a 10-item short form suitable for when time and survey length are constrained. We invite interested researchers to download the raw data, analysis files, and copies of the long and short forms from Open Science Framework (https://osf.io/d9ujk/).

### Study 1: Development of the 36-item scale

In Study 1, we developed and evaluated the long form of the Revised Aesthetic Fluency Scale. After describing the development and refinement of the response scale and initial item pool, we present a detailed psychometric evaluation of the 36-item scale along with some initial evidence for the validity of its scores.

### Method

#### Participants

This research was approved by the University of North Carolina Institutional Review Board (Approval #21–0057). All participants provided written informed consent. The psychometric analyses were based on a final sample of 2089 English-speaking adults—1044 women, 1045 men—who took part via the Prolific.co survey panel. The participants ranged widely in age (*M* = 33.16 years, *SD* = 12.57, *Mdn* = 30, range from 18 to 92) and were predominantly located in the USA, UK, Canada, and Australia. This final sample was whittled from a slightly larger initial sample (*n* = 2230; 6.3% excluded) after applying exclusions for likely careless and inattentive responding—such as extensive missing data, missed directed response items, unusually long strings of item responses, unusually short survey times, and elevated Mahalanobis *D* values reflecting unusual response patterns [25, 26]—using the R package *careless* 1.21 [27]. The study was not preregistered.

#### Procedure

The process of scale development began by considering the intended population. Because the original Aesthetic Fluency Scale measures art knowledge most reliably among very knowledgeable respondents [21]—we wanted to develop the scale to be fruitful in research with samples that vary widely in their art knowledge, from experts to novices with relatively little background, knowledge, or interest in the arts. We also wanted the scale to be useful in field settings (e.g., researchers working in museums, galleries, and heritage sites) and in lab and online studies.

We started by creating a bank of 153 possible items. The authors collectively brainstormed an item pool that was guided by the goals of having a wide range of difficulty (i.e., both well-known and obscure items); good representation of periods, styles, and genre; diversity in the artists; and items related to artistic materials, tools, and techniques. An early decision was made to focus the items primarily on the Western art tradition because we anticipated that most of the researchers using the scale would be assessing Western populations, and because the scope and richness of non-Western traditions call for versions of the scale that are specifically developed to measure knowledge of them. The targeted length of the final scale was around 40 items, which struck us as a good balance of content coverage and survey duration.

As part of the development process, we revisited the original scale’s 5-point response format. The wording for the response options was simplified, and the response options were condensed into three options:

- 0 = I don’t really know anything about this artist or term
- 1 = I’m familiar with this artist or term
- 2 = I know a lot about this artist or term

#### Refining the item pool

We first eliminated 40 items from the original pool of 153 based on a priori grounds, such as items that were far too obscure (and thus would be too “difficult” in IRT terms), items that could potentially be confused with similar terms outside of the arts, or overlapping items. Data collection then proceeded in several waves using the Prolific.co research participant pool. To broaden the range of likely art knowledge in our sample, we took advantage of Prolific’s ability to select participants based on their hobbies and backgrounds. In each sample, around 20% of the participants had indicated “art” as one of their main hobbies, another 20% indicated past employment in the arts sector, and the remaining participants were from the broader, unselected participant pool. Note that our goal was simply to expand the potential variance of art knowledge in the sample, and that these aren’t distinct, exclusive groups—many participants in the unselected pool, for example, surely had art as a hobby or had worked in the arts sector.

For the first wave, we collected responses from 513 participants for 113 items. Items were dropped based on disordered item thresholds [24] and extreme item difficulty. For the second wave, a sample of 499 people responded to 77 remaining items. Responses to those items from the first wave were added, so the 77 items were evaluated with a sample of 1012 people. Items were again dropped based on disordered thresholds and extreme item difficulty. In addition, unique variable analysis [28] was used to illuminate clusters of redundant items, which allowed us to trim related items that created minor factors.

For the third and final wave, a sample of 1090 people responded to 51 remaining items. The prior waves’ responses were added to create a large final sample. The pool of 51 items was whittled to the final set of 36 items using many psychometric criteria, such as emphasizing essential unidimensionality, avoiding high local dependence between items, evaluating IRT item fit metrics like Outfit, Infit, and RMSD [22, 29], and avoiding differential item functioning by gender. In addition, we used substantive and subjective criteria for the final decisions, such as ensuring good representation of women artists, avoiding terms with salient meaning outside of the arts (e.g., Baroque), and balancing the distribution of items from various periods and countries.

#### Initial evidence for score validity

To obtain initial evidence for score validity, all the samples completed a handful of additional items related to their background and interest in the arts. We included two items related to art interest from the VAIAK [9], which were completed on a 5-point scale (1 = *strongly disagree*, 5 = *strongly agree)*:

- I am interested in art.
- I enjoy talking about art with others.

Several other items asked about people’s past background in the arts, using a binary scale (0 = *No*, 1 = *Yes*):

- Have you ever taken a course in art, design, or art history?
- Have you ever had a job involving visual art, design, or art history?
- In the past 5 years, have you created an original work of visual art or design?
- In the past 5 years, have you shared your artwork or artistic ideas in a public space (real or virtual)?
- In the past 5 years, have you visited a gallery or museum to view or learn about art?

### Results

The psychometric analyses were done in R 4.2 [30] using the packages *TAM* 4.1.4 [31], *psych* 2.2.5 [32], and *EGAnet* 1.2.3 [33]. Effect sizes are presented in the Pearson *r* metric (using *r* = 0.10, 0.30, and 0.50 values as benchmarks for small, medium, and large effects) for continuous variables and Cohen’s *d* (using *d* = 0.20, 0.50, and 0.80 as benchmarks) for categorical predictors. All confidence intervals (in brackets) are 95%.

#### Psychometric properties

We report the psychometric properties of the 36-item Revised Aesthetic Fluency Scale using the responses to these items from all three waves. Because analyses of gender-based item bias require large group sizes, a handful of participants (*n* = 13) who did not indicate their gender identity or identify as male or female were omitted, leaving a final sample of 2089 participants. As noted earlier, this sample was evenly balanced between men and women (1045 men, 1044 women) and had a wide age range (*M* = 33.16 years, *SD* = 12.57, *Mdn* = 30).

**Fig 2:**
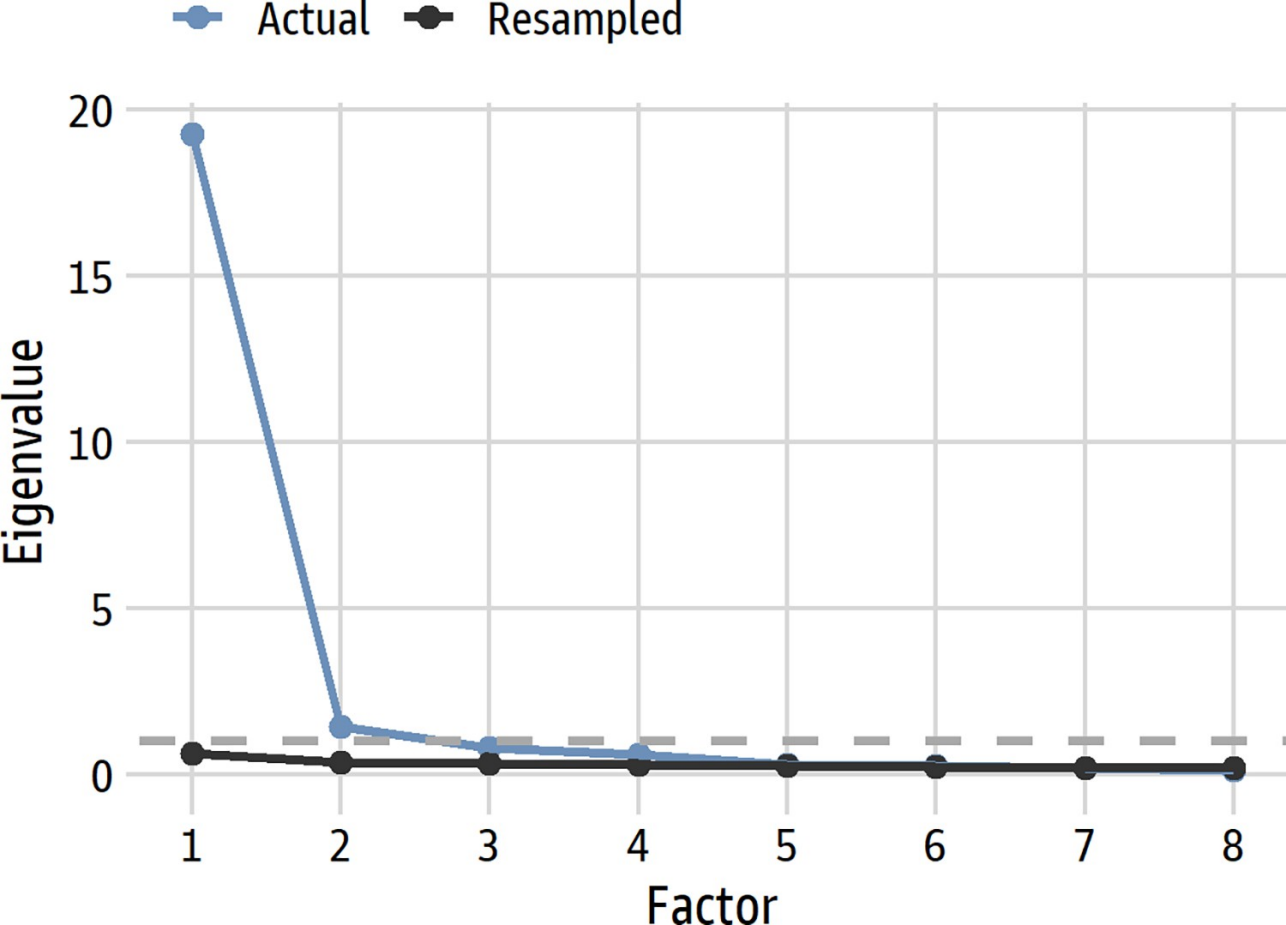
Parallel analysis scree plot for the 36 items. The dashed line denotes an eigenvalue of 1. For clarity, only the first 8 factors are shown.

*Internal consistency and unidimensionality*. Analyses of internal consistency and dimensionality suggested that the 36-item scale was essentially unidimensional [34], a looser criterion than strict unidimensionality that recognizes that self-report scales often have trivial minor factors. Factor analyses, using maximum likelihood and polychoric correlations, supported unidimensionality. A parallel analysis [35] suggested six factors but clearly revealed a single dominant one (see Fig 2). The first eigenvalue (19.23) was over 13 times greater than the second (1.44), which greatly exceeds the guidelines of 3:1 and 4:1 as indicators of essential unidimensionality [34]. An exploratory factor analysis with a bifactor rotation [36] evaluated how well each item loaded on a general common factor as well as specific factors, if any. Six factors were explored (one general and five specific) following the parallel analysis. All items loaded higher on the general factor than on any specific factor (loadings ranged from 0.55 to 0.83 on the general factor), and only a handful of loadings on the specific factors exceeded 0.40. Regarding internal consistency, Cronbach’s α was 0.96, and ω-hierarchical was 0.92. Taken together, the analyses support the view of the scale as essentially unidimensional.

*IRT model selection*. The item response analyses used a generalized partial credit model [37], which fit the data better than a simpler partial credit model according to information-theory metrics. This IRT model estimates an item difficulty parameter (*b*), an item discrimination parameter (*a*), and two item thresholds that reflect the boundary points between the 0:1 and 1:2 response options. The models were estimated using marginal maximum likelihood with Ramsay acceleration. The model was identified via case constraints, so a latent trait score of zero represents the mean estimated latent trait score in the sample. The reliability of the estimated EAP (expected a posteriori) trait score was 0.94.

**Fig 3:**
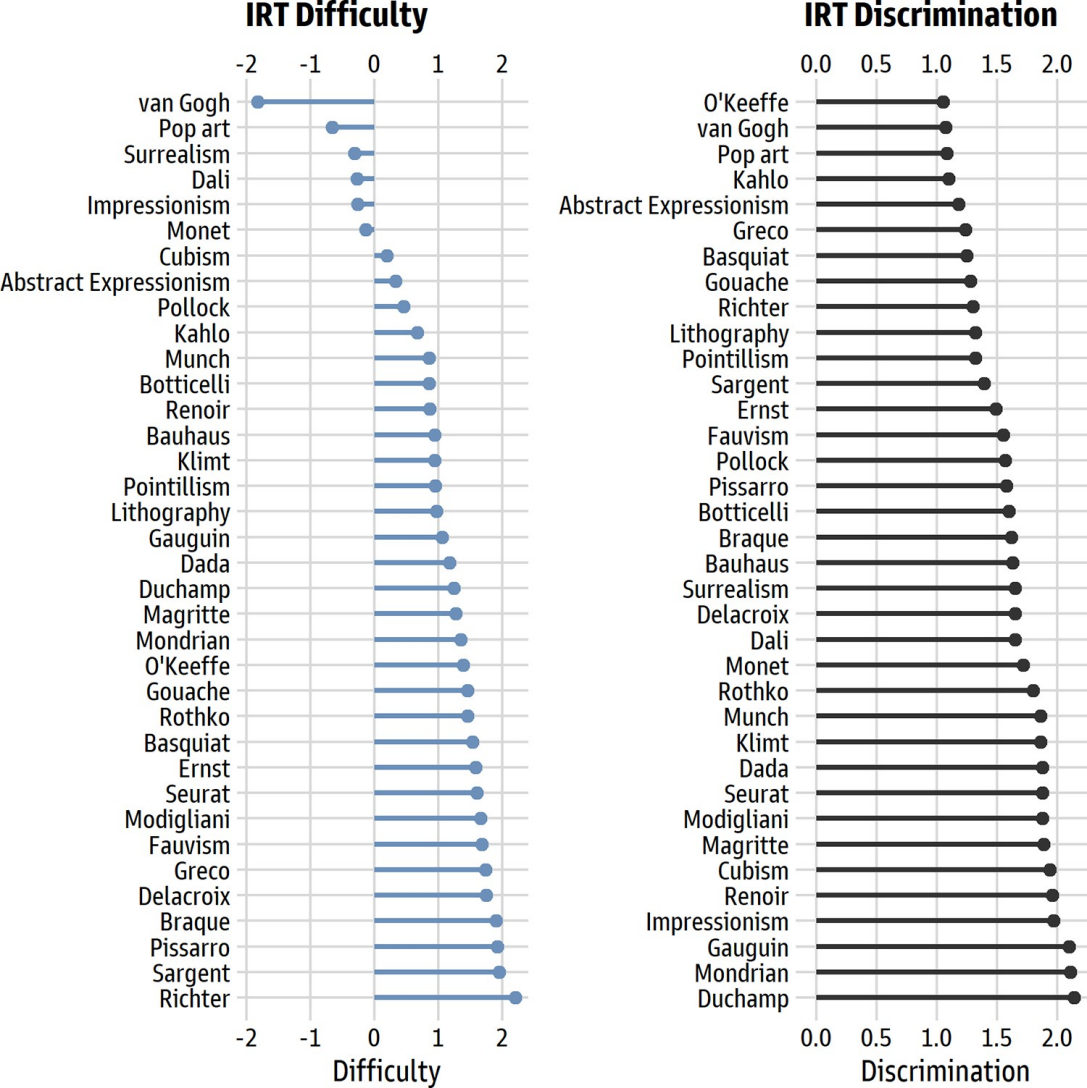
IRT difficulty and discrimination values for the 36 items. The left panel shows the values for the IRT difficulty parameter; the right panel shows the values for the IRT discrimination parameter.

*Item difficulty and discrimination*. As intended, the items showed a wide range of difficulty, from -1.82 (Vincent van Gogh, the easiest item) to 2.20 (Gerhard Richter, the hardest). The item discrimination values ranged from 1.05 to 2.14, so all items had strong links to the underlying trait (see Fig 3).

*Threshold behavior*. All 36 items had properly ordered thresholds, so each response option had a region of the underlying trait where it was the most likely response [24]. Table 1 displays the logit-unit space between the two thresholds for each item.

**Table 1:** Revised Aesthetic Fluency Scale: Items and statistics.

Item	*M (SD)*	IRT *a* (discrimination)	IRT *b* (difficulty)	Threshold distance	RMSD Item Fit	Infit	Outfit
Gerhard Richter	0.15 (0.43)	1.30	2.20	0.44	.018	1.01	0.91
John Singer Sargent	0.24 (0.49)	1.39	1.95	1.35	.020	1.02	0.91
Alessandro Botticelli	0.60 (0.66)	1.60	.85	1.74	.026	1.01	0.99
Jean-Michel Basquiat	0.32 (0.59)	1.25	1.53	0.66	.015	1.00	1.00
Frida Kahlo	0.68 (0.76)	1.10	.67	0.86	.031	1.00	1.02
Claude Monet	1.04 (0.67)	1.72	-0.14	2.03	.016	1.00	0.99
Georgia O’Keeffe	0.44 (0.64)	1.05	1.39	1.12	.029	1.00	1.00
Jackson Pollock	0.77 (0.70)	1.57	0.46	1.72	.017	1.00	0.99
Piet Mondrian	0.29 (0.58)	2.11	1.35	0.77	.021	1.02	1.02
Salvador Dalí	1.10 (0.70)	1.65	-0.27	1.84	.016	1.00	1.00
Vincent van Gogh	1.45 (0.55)	1.07	-1.82	3.66	.026	1.01	0.99
Marcel Duchamp	0.35 (0.60)	2.14	1.24	1.07	.020	1.00	1.09
Georges Braque	0.19 (0.46)	1.62	1.90	0.85	.012	0.98	1.07
Eugène Delacroix	0.25 (0.50)	1.65	1.75	1.19	.017	1.01	1.29*
El Greco	0.34 (0.55)	1.24	1.74	1.63	.020	1.01	1.00
Max Ernst	0.31 (0.56)	1.49	1.58	1.23	.018	0.98	1.10
Paul Gauguin	0.43 (0.64)	2.10	1.06	1.17	.018	1.01	1.00
Gustav Klimt	0.48 (0.69)	1.86	0.94	0.89	.016	1.00	1.01
René Magritte	0.35 (0.61)	1.89	1.27	0.92	.022	1.02	0.95
Amedeo Modigliani	0.23 (0.50)	1.88	1.66	0.90	.029	1.06	0.88
Edvard Munch	0.55 (0.68)	1.86	0.85	1.34	.022	1.00	1.04
Camille Pissarro	0.21 (0.47)	1.58	1.92	1.09	.019	1.00	1.19
Pierre-Auguste Renoir	0.56 (0.66)	1.96	0.86	1.51	.031	1.01	0.94
Mark Rothko	0.28 (0.57)	1.80	1.45	0.60	.028	1.02	1.02
Georges Seurat	0.24 (0.52)	1.88	1.60	0.78	.010	1.01	1.01
Fauvism	0.26 (0.53)	1.55	1.68	0.99	.016	0.99	0.96
Impressionism	1.08 (0.61)	1.97	-0.26	2.33	.024	1.00	0.97
Abstract Expressionism	0.87 (0.64)	1.18	0.33	2.62	.024	1.00	1.00
Cubism	0.89 (0.66)	1.94	0.19	2.00	.014	1.00	0.98
Dada	0.40 (0.63)	1.88	1.17	1.09	.024	1.03	0.92
Pointillism	0.54 (0.70)	1.32	0.95	1.01	.022	1.00	1.02
Pop art	1.18 (0.61)	1.08	-0.66	3.03	.021	1.00	0.99
Surrealism	1.10 (0.63)	1.65	-0.31	2.32	.023	1.00	0.99
Bauhaus	0.52 (0.68)	1.63	0.94	1.23	.023	1.00	1.04
Gouache	0.37 (0.61)	1.28	1.45	0.98	.030	1.02	0.99
Lithography	0.59 (0.65)	1.32	0.97	1.94	.017	1.00	1.00

The items are presented in a different random order for each participant. A research-ready Qualtrics version can be downloaded from Open Science Framework (https://osf.io/d9ujk/). The response scale ranges from 0 to 2. Threshold distance refers to the distance (in logits) between the two response thresholds. Lower RMSD values indicate better item fit. For Infit and Outfit, 1 represents ideal fit. Only one item (Eugène Delacroix) had a statistically significant Outfit value.

**Fig 4:**
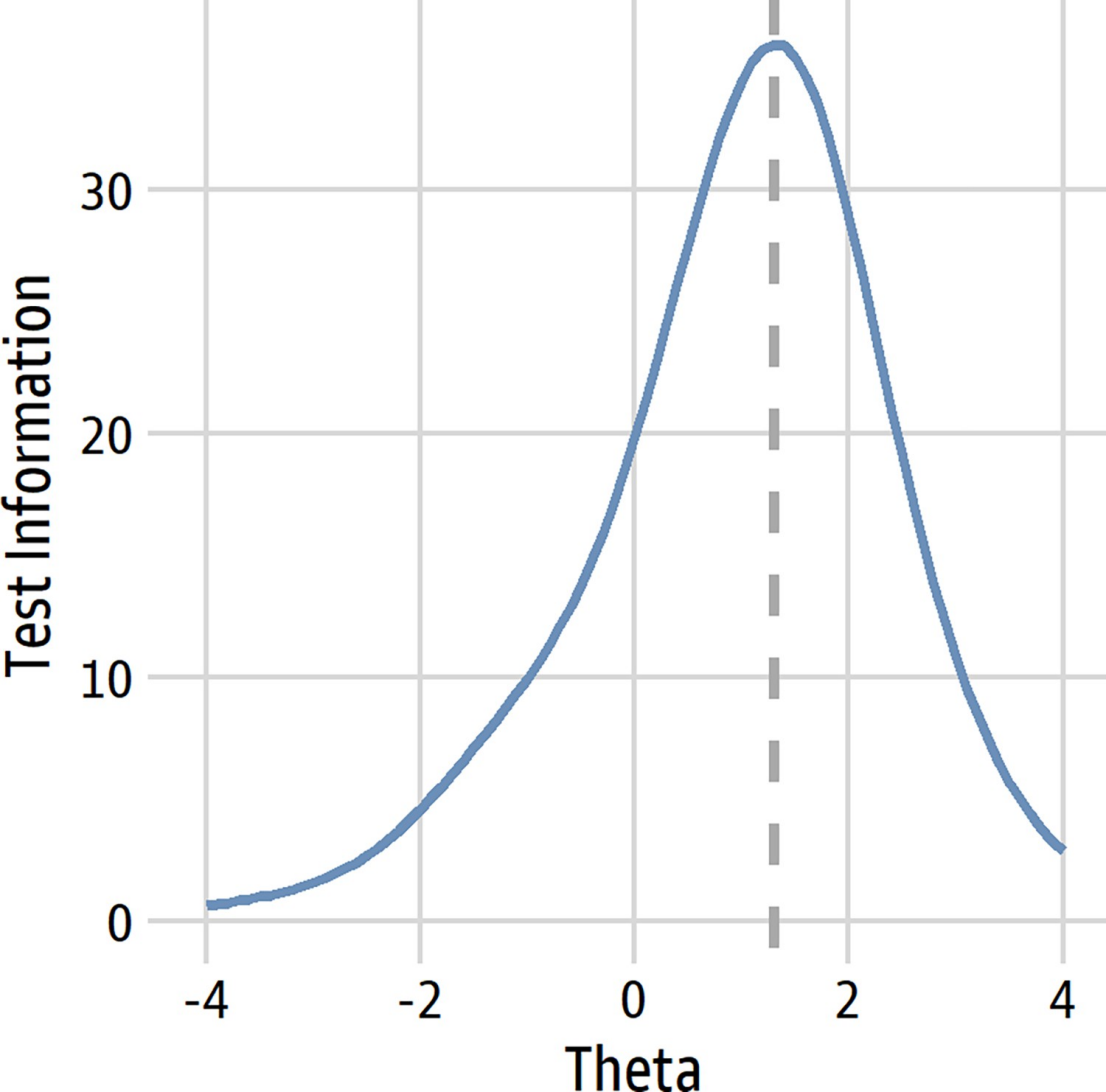
Test information function. The dashed line denotes the peak at 1.30.

*Overall test information*. The test information function shows the regions of the underlying trait for which the scale provides the most information (i.e., where it estimates people’s trait scores with the least error). The test information function peaked at 1.30 (Fig 4), so it is most reliable at ordering fairly knowledgeable participants relative to each other. This peak is much lower than the peak of the original scale [21], so the revised scale is relatively easier, as we intended.

*Item fit*. Item fit was assessed with Infit and Outfit values [22, 38] and with RMSD [29]. For Infit and Outfit, the values were close to 1 for most items and within 0.80 to 1.30 for all items. Only 1 item (Eugène Delacroix) had a statistically significant Outfit value (see Table 1). For RMSD, misfit is judged as “negligible” for values less than 0.02, “small” for values between 0.02 and 0.05, and “medium” for values between 0.05 and 0.08 reflected “medium” misfit. The highest RMSD values were only 0.031 (Frida Kahlo and Pierre-Auguste Renoir), so the items as whole showed modest misfit.

*Local dependence*. Local dependence between item pairs was evaluated with the adjusted *Q*_3_ statistic (a*Q*_3_), a corrected version of Yen’s *Q*_3_ that centers the values on the average *Q*_3_ score [39, 40]. No item pairs had values greater than 0.30, and eight pairs had values over 0.20. The largest values were for item pairs that were culturally popular or conceptually similar (e.g., van Gogh and Pop Art, a*Q*_3_ = 0.26; Abstract Expressionism and Impressionism, a*Q*_3_ = 0.26).

*Differential item functioning*. Finally, we evaluated differential item functioning (DIF), which occurs when members of different groups have the same trait level yet different probabilities of giving a particular response [41]. In such cases, the probability of an item response is not solely due to the respondent’s trait level, as it should be, but is also affected by construct-irrelevant factors [42]. Using the R package *lordif* 0.3.3 [43, 44], DIF was evaluated in terms of effect sizes for gender and for past employment in the arts. Small effect sizes in the *R*^2^ metric (2% of the variance using McFadden’s *R*^2^) were flagged for possible DIF.

For gender (1044 women, 1045 men), only one item showed DIF. The “Frida Kahlo” item had a small DIF effect (*R*^2^ = 3.74%) that favored women: for women and men with equal underlying art knowledge, women are nevertheless slightly more likely to endorse knowing more about Frida Kahlo. We ultimately decided to retain the item to balance the low DIF with retaining relatively more items related to women artists. For past employment in the arts, around 20% of the sample had held an art job (*n* = 408 of the total 2066 responses). No items were flagged for DIF based on past employment, so differences in the scale scores for these groups likely reflect true trait differences.

#### Descriptive statistics for aesthetic fluency scores

**Fig 5:**
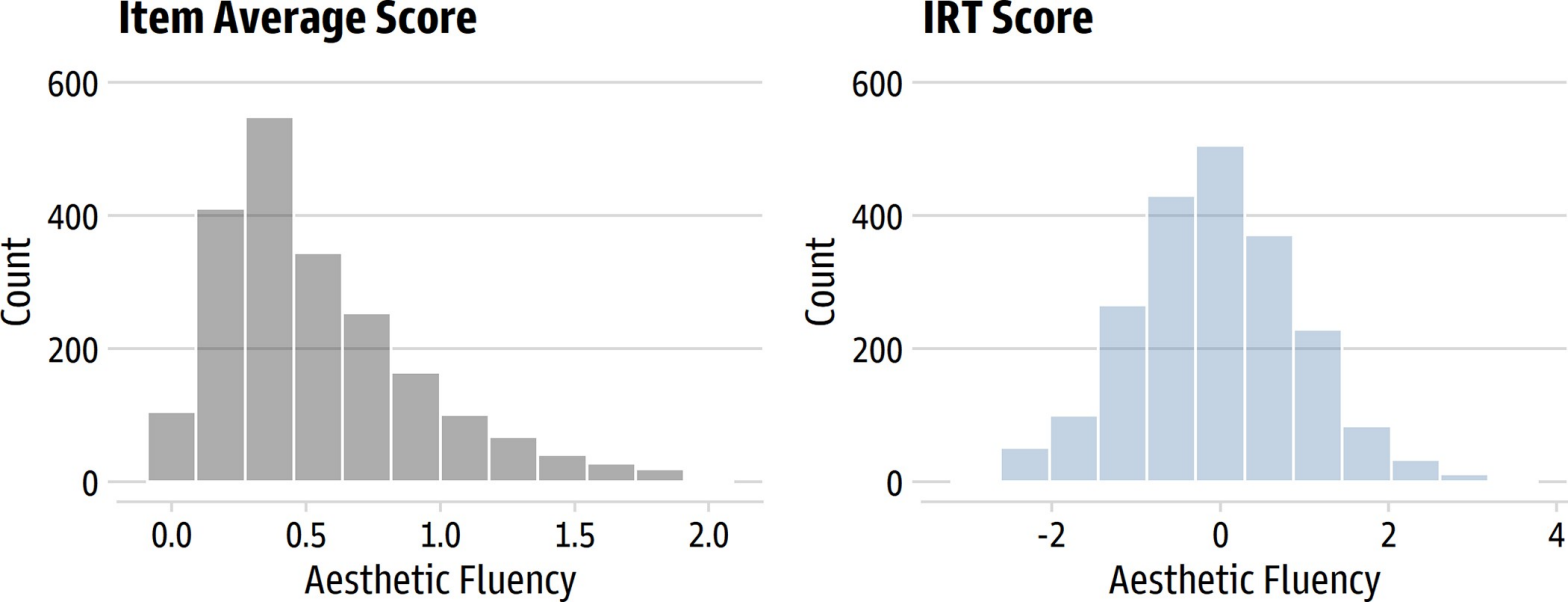
Distribution of average and IRT-based aesthetic fluency scores. The left panel shows the average of the scale’s items; the right panel shows the trait score estimated from the IRT model.

Fig 5 displays the distribution of averaged and IRT-based aesthetic fluency scores. Like the original scale, the item average was positively skewed (*M* = 0.55, *SD* = 0.38, *Mdn* = 0.44, range from 0 to 2); the IRT-based trait score was relatively normal (*M* = -0.05, *SD* = 1.01, *Mdn* = -0.04, range from -2.69 to 3.70).

#### Relationships with demographic and art engagement variables

**Fig 6:**
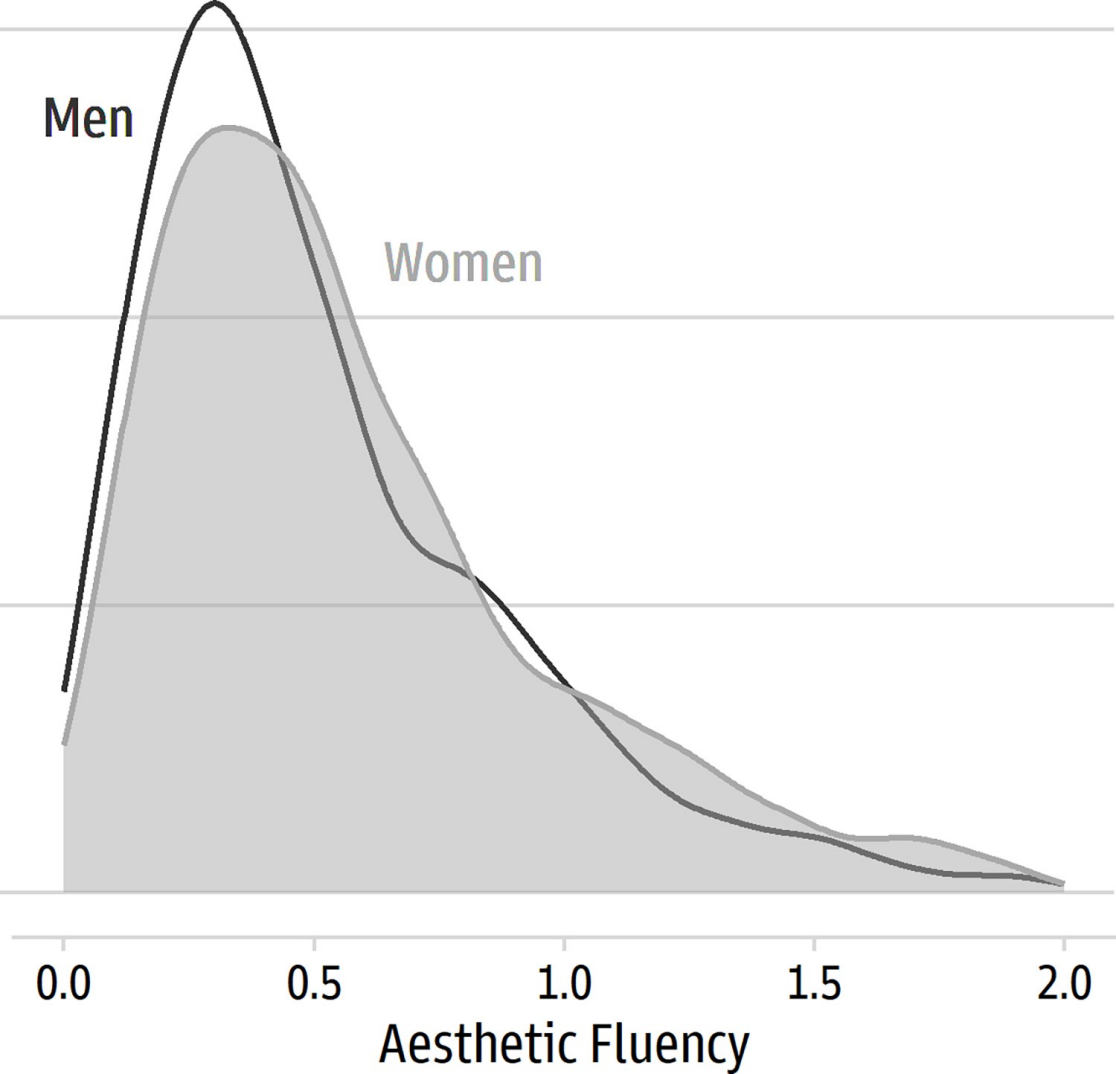
Distribution of average aesthetic fluency scores for women and men.

We explored some relationships using the averaged item scores. Age had a modest correlation with aesthetic fluency (*r* = 0.16 [0.12, 0.21], *p* < 0.001); older members of the sample had higher scores. Likewise, gender had a small effect, in Cohen’s *d* terms (*d* = 0.16 [0.08, 0.25]), due to higher scores for women than men (see Fig 6).

**Fig 7:**
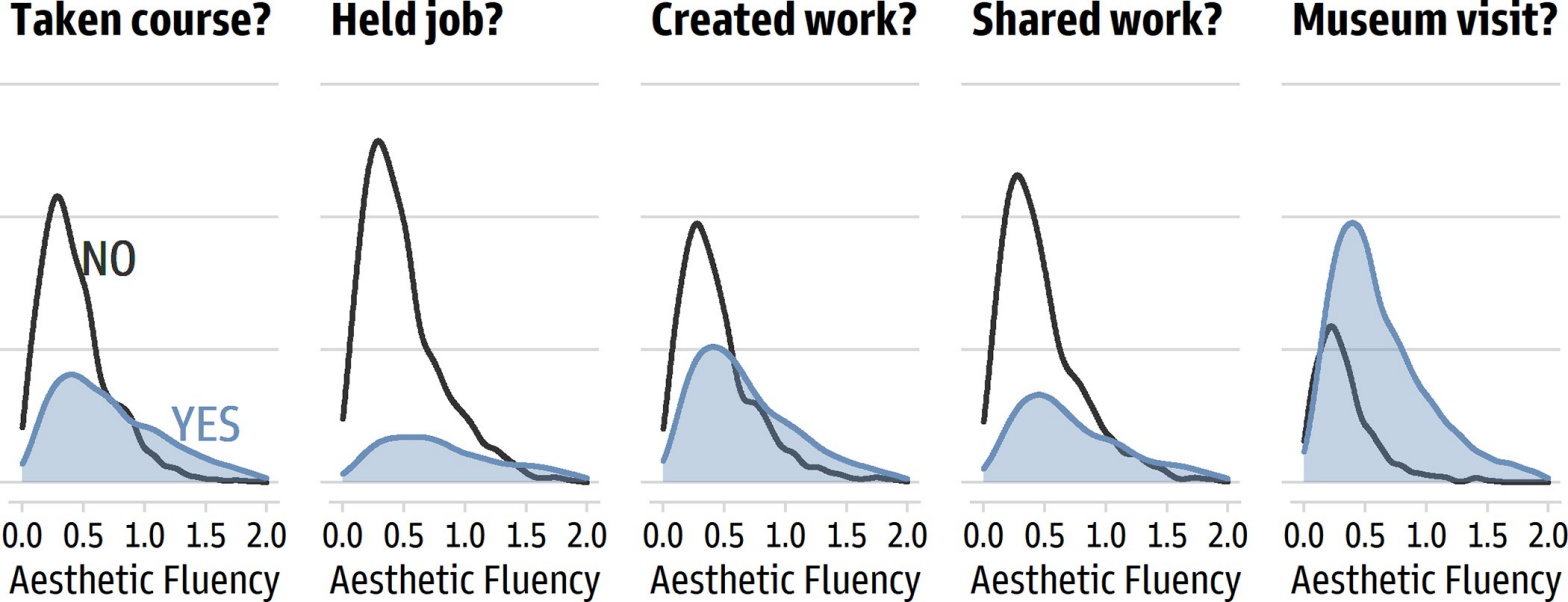
Differences in average aesthetic fluency scores based on art background and engagement.

As expected, the items about people’s background and engagement in the arts found higher aesthetic fluency scores among people who said they had taken a course in art, design, or art history (*d* = 0.78 [0.69, 0.87]); who had worked in a job involving visual art, design, or art history (*d* = 0.90 [0.79, 1.01]); created an original work of visual art or design in the past five years (*d* = 0.60 [0.51, 0.69]); shared artwork in a public space in the past five years (*d* = 0.68 [0.58, 0.77]); and visited a gallery or museum in the past five years (*d* = 0.78 [0.67, 0.88]). Fig 7 displays the distributions. Finally, the two VAIAK items related to interest in the arts, as expected, covaried with aesthetic fluency scores. Large correlations were found for “I am interested in art” (*r* = 0.54 [0.51, 0.57]) and for “I enjoy talking about art with others” (*r* = 0.55 [0.52, 0.58]).

## Discussion

Taken together, the psychometric qualities of the Revised Aesthetic Fluency Scale suggest that it is a promising addition to an arts researcher’s self-report toolbox. Evidence for dimensionality points to an essentially unidimensional scale with minimal secondary factors and with high internal consistency reliability. IRT analyses indicate that the scale’s items are well-behaved: they have ordered thresholds, high discrimination levels, the desired range of item difficulty, and good fit to the IRT model based on item-fit metrics. Finally, analyses of differential item functioning suggested at most minimal item bias for gender (women had a slight edge for the “Frida Kahlo” item) and no item bias for past employment for the arts. Researchers can thus be confident that observed differences as a function of these variables reflect substantive underlying differences in the construct of interest, not secondary or nuisance factors.

As part of the scale’s development, we included some additional items to evaluate the low-hanging fruit of evidence for score validity: people’s interest in art and their past engagement with it. As one would expect, people who reported greater art interest (measured with a pair of VAIAK items) had higher aesthetic fluency scores, and people with greater engagement in the arts (e.g., working in the arts or visiting museums) had higher aesthetic fluency scores. These results represent the sorts of findings one would expect to find if the scale measured its intended construct.

## Study 2: Evidence for score validity

In Study 2, we sought additional evidence for the score validity of the Revised Aesthetic Fluency Scale using a new, independent sample of adults. To explore validity, we focused on measures of individual differences that should covary with knowledge of the arts. First, participants completed a measure of the Big Five factors of personality. Although all five factors have their roles in the psychology of the arts [45], Openness to Experience looms large as a predictor of people’s engagement with the arts, both as audiences and observers [46] and as creators [47]. Notably, Openness to Experience consistently predicted scores on the original scale [11, 13, 48], so finding similar relationships with the new scale would provide unsurprising but important information. Second, participants completed the Aesthetic Responsiveness Assessment (AReA) [49], a self-report scale that measures three facets of people’s emotional and behavioral engagement with art. Taken together, the study affords a look at whether individual differences in the Revised Aesthetic Fluency Scale covary as they should with constructs connected to art engagement.

### Method

#### Participants

This research was approved by the University of North Carolina Institutional Review Board (Approval #21–0057). All participants provided written informed consent. The final sample consisted of 392 English-speaking adults—199 women, 193 men—who were recruited through the Prolific.co participant pool. The respondents ranged in age from 18 to 76 (*M* = 37.43, *SD* = 13.30, *Mdn* = 35) years and were primarily from the USA, UK, Canada, and Australia. The final sample comes from slightly larger sample (*n* = 424; 7.5% excluded) that had been filtered based on markers of careless and inattentive responding. As before, we oversampled participants who indicated that art was a major hobby and who indicated having worked in the arts sector.

#### Procedure

Participants completed a survey via Qualtrics that included the 36-item Revised Aesthetic Fluency Scale (α = 0.95), demographic items, and two measures of individual differences relevant to aesthetics and the arts.

For individual differences specific to aesthetics, we included the Aesthetic Responsiveness Assessment (AReA) [49], a 14-item self-report scale that measures three facets of how people respond to art: *aesthetic appreciation* (8 items; α = 0.90), *intense aesthetic experience* (4 items; α = 0.83), and *creative behavior* (3 items; α = 0.73). The aesthetic appreciation facet emphasizes behavioral engagement (“I visit museums or go to musical/dance performances”) and emotional engagement (“I appreciate the visual design of buildings”) with the arts. The intense aesthetic experience facet emphasizes strong emotional reactions to the arts, typically sublime and awe-like experiences (“I experience awe, fear, or a feeling of being overwhelmed when looking at art”). Finally, a small creative behavior facet emphasizes engaging in artistic actions (“I write poetry or fiction”). Note that a quirk of the AReA that is one item (“I am deeply moved when I see art”) is included in both the aesthetic appreciation and intense aesthetic experience facets. Participants responded to each item using a 5-point scale (0 = *Never*, 4 = *Very often*).

In addition, we measured the Big 5 personality factors with the 30-item BFI-2 [50], which measures Neuroticism (α = 0.85), Extraversion (α = 0.78), Openness to Experience (α = 0.82), Agreeableness (α = 0.77), and Conscientiousness (α = 0.79) with six items per factor using a 5-point response scale (1 = *disagree strongly*, 5 = *agree strongly*). Openness to Experience has strong links to people’s level of art interest, knowledge, and engagement—including the original Aesthetic Fluency Scale [13]—so one would expect this trait to strongly covary with scores on the revised scale. There were no specific expectations for the other four traits. The study was not preregistered.

#### Results and discussion

**Fig 8:**
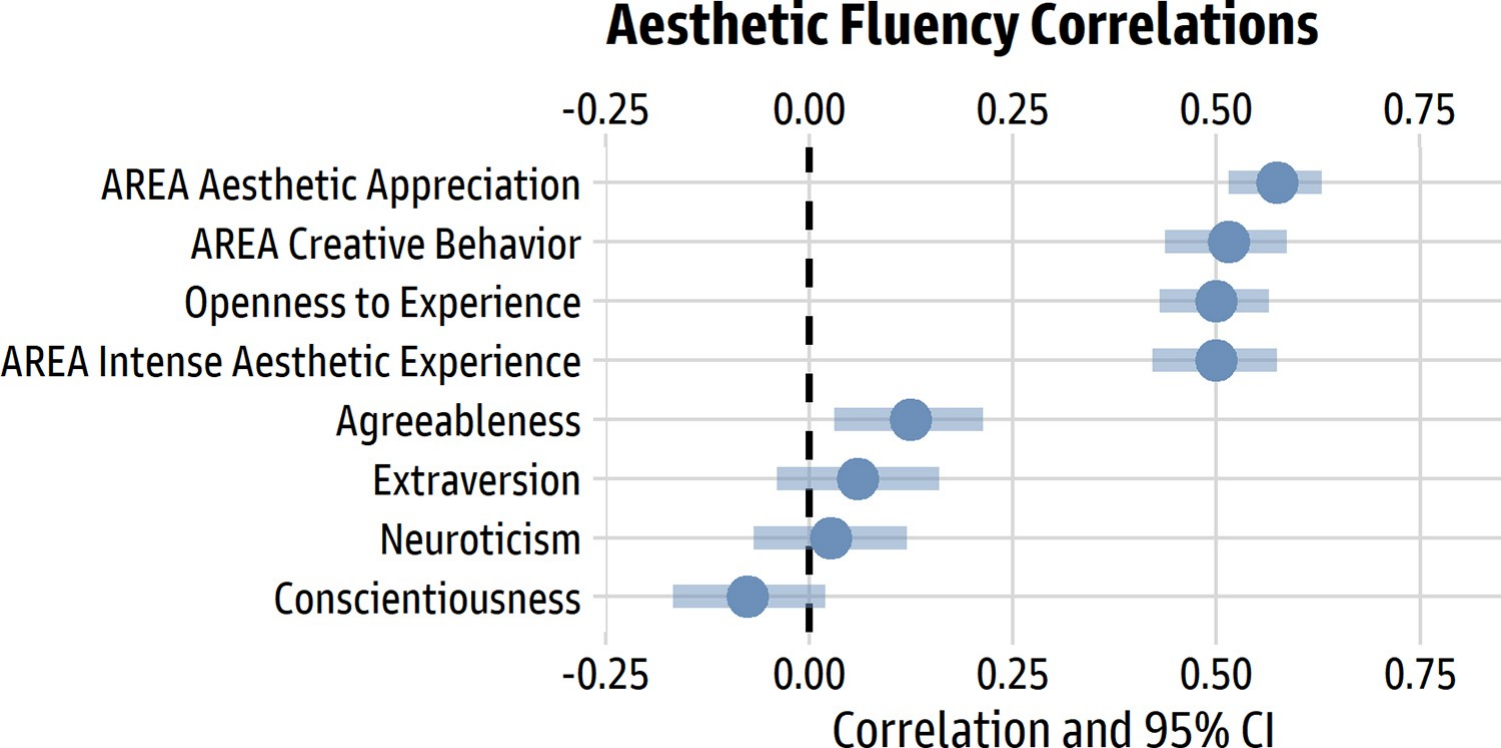
Pearson correlations for the revised Aesthetic Fluency Scale with big 5 traits and AReA subscales. The dots represent the Pearson *r* correlation value; the bars represent the 95% confidence interval around *r*.

Table 2 shows descriptive statistics and correlations for all the variables, which were formed via item averages, and Fig 8 illustrates the zero-order Pearson correlations between scores for the Revised Aesthetic Fluency Scale and the other measures of personality and individual differences. It is apparent that the AReA subscales and Openness to Experience strongly covary with aesthetic fluency.

**Table 2:** Descriptive statistics and correlations: Study 2.

Variable	*M*	*SD*	*Mdn*	1	2	3	4	5	6	7	8	9
1 Aesthetic Fluency	0.53	0.33	0.44	1								
2. AREA Aesthetic Appreciation	2.27	0.79	2.38	0.57	1							
3. AREA Intense Aesthetic Experience	1.29	0.82	1.25	0.50	0.83	1						
4. AREA Creative Behavior	1.10	1.02	1.00	0.52	0.60	0.58	1					
5. Neuroticism	2.99	0.92	3.00	0.03	0.05	0.12	0.19	1				
6. Extraversion	2.94	0.80	2.83	0.06	0.15	0.10	0.05	-0.39	1			
7. Openness to Experience	3.64	0.84	3.67	0.50	0.72	0.58	0.64	-0.01	0.19	1		
8. Agreeableness	3.75	0.71	3.83	0.12	0.29	0.21	0.13	-0.27	0.24	0.24	1	
9. Conscientiousness	3.48	0.78	3.50	-0.08	-0.02	-0.05	-0.10	-0.31	0.22	0.03	0.28	1

*n* = 392.

Regression models, estimated in R using robust standard errors and standardized regression weights via the package *parameters* 0.18.2 [51], estimated the unique effects of the three AReA subscales (model *R*^2^ = 38%). The largest effects were for aesthetic appreciation (β = 0.41 [0.26, 0.56], *p* < 0.001) and creative behavior (β = 0.27 [0.15, 0.38], *p* < 0.001); the unique effect for intense aesthetic experience was essentially zero (β = 0.01 [-0.16, 0.18], *p* = 0.909).

Likewise, for the Big 5 personality traits, the unique effects for each trait were estimated in a similar model (model *R*^2^ = 26%). Only Openness to Experience had a large effect (β = 0.50 [0.41, 0.59], *p* < 0.001); small and near-zero effects were found for Neuroticism (β = 0.00 [-0.09, 0.10], *p* = 0.959), Extraversion (β = -0.02 [-0.11, 0.07], *p* = 0.614), Agreeableness (β = 0.04 [-0.05, 0.13], *p* = 0.409), and Conscientiousness (β = -0.10 [-0.19, -0.01], *p* = 0.031).

Taken together, the findings support intuitive expectations for what people high in aesthetic fluency are like. The largest effect size was for Openness to Experience, a central trait in the psychology of the arts, followed by AReA aesthetic appreciation (behavioral and emotional engagement with the arts) and AReA creative behavior (engaging in art making).

## Study 3: Developing a brief form

The original Aesthetic Fluency Scale was grounded in field research in museums and galleries [6], where long surveys are impractical because of a need to balance visitors’ time and goodwill with the scholarly aims of the research. To serve researchers seeking a crisp, brief scale, we honed the 36-item Revised Aesthetic Fluency Scale into a 10-item version suitable for when time and survey length are limited.

### Method

#### Participants

The participants from Studies 1 and 2 were pooled to create a large sample for evaluating items for inclusion in a short form. After omitting one case with extensive missingness and cases that did not identify as male or female (for gender-based DIF analysis purposes), this resulted in a sample size of 2480 (1242 women, 1238 men).

#### Procedure

We selected items for the short form using a combination of quantitative criteria and content aims. Reflexively applying quantitative rules or processes (e.g., selecting items with the highest factor loadings or using automated selection methods) can yield short forms with restricted content domains or unwanted item overlap. Our goal was to create a short form with a similar test-information peak so that the long and short forms are similarly difficult and provide the most information around the same region of the underlying trait. This entailed sorting the 36 items by difficulty and selecting items that covered the full difficulty range, with the constraint of a TIF peak that approximated 1.30. Item selection was further guided by sensible content constraints. We wanted reasonable coverage of the diversity of items in the full scale (e.g., artists, styles, and tools in different periods) and minimal conceptual overlap between the items (e.g., avoiding having both “Pop art” and “Andy Warhol” or both “Impressionism” and “Claude Monet”). Finally, we excluded the only DIF item (“Frida Kahlo”) from consideration in the short form because it would carry relatively more weight in a 10-item scale.

After evaluating and modifying many 10-item sets of items, based on quantitative metrics and our domain knowledge, we settled on a short form of 10 items: Pop art, Claude Monet, Cubism, Alessandro Botticelli, Gustav Klimt, Lithography, Gouache, Georgia O’Keeffe, Jean-Michel Basquiat, and Amedeo Modigliani.

### Results and discussion

**Fig 9:**
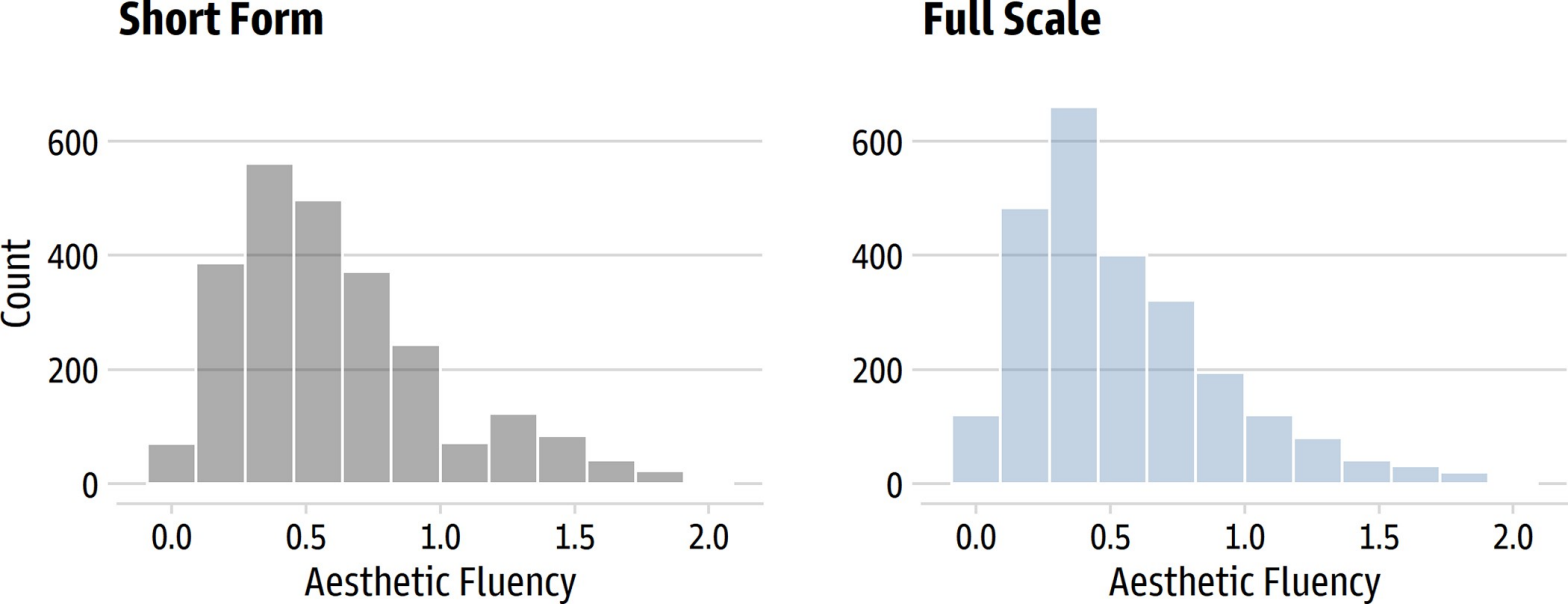
Distribution of average scores for the short form and full scale. The left panel shows the 10-item short form scores; the right panel shows the 36-item full scale scores.

We evaluated the short form using the same psychometric methods described in Study 1. The short form showed strong evidence for unidimensionality. The first eigenvalue (4.89) was over 15 times larger than second (0.31) in a parallel analysis, which suggests essential unidimensionality [34]. The distributions of the averaged item scores for the short form and full scale for the full sample of 2480 people are shown in Fig 9. The Spearman’s rank-order correlation between the long and short forms was very high (ρ = 0.95 [0.94, 0.95]), so participants tended to be in the same position relative to each other regardless of the form. Cronbach’s α was 0.84 for the short form and 0.96 for the full scale; ω-hierarchical was 0.75 for the short form and 0.91 for the full scale.

A generalized partial credit model indicated that, as intended, the test information function peaked at the same region as the full scale (1.30). All thresholds were ordered, item fit was good based on RMSD (items ranged from 0.012 to 0.030) and Infit and Outfit (no significant misfit), and all a*Q*_3_ values were under |0.16|. No items were flagged for gender-based DIF. Small gender differences favoring women were found for both the short form (*d* = 0.25 [0.17, 0.33]) and full scale (*d* = 0.16 [0.08, 0.24]).

**Fig 10:**
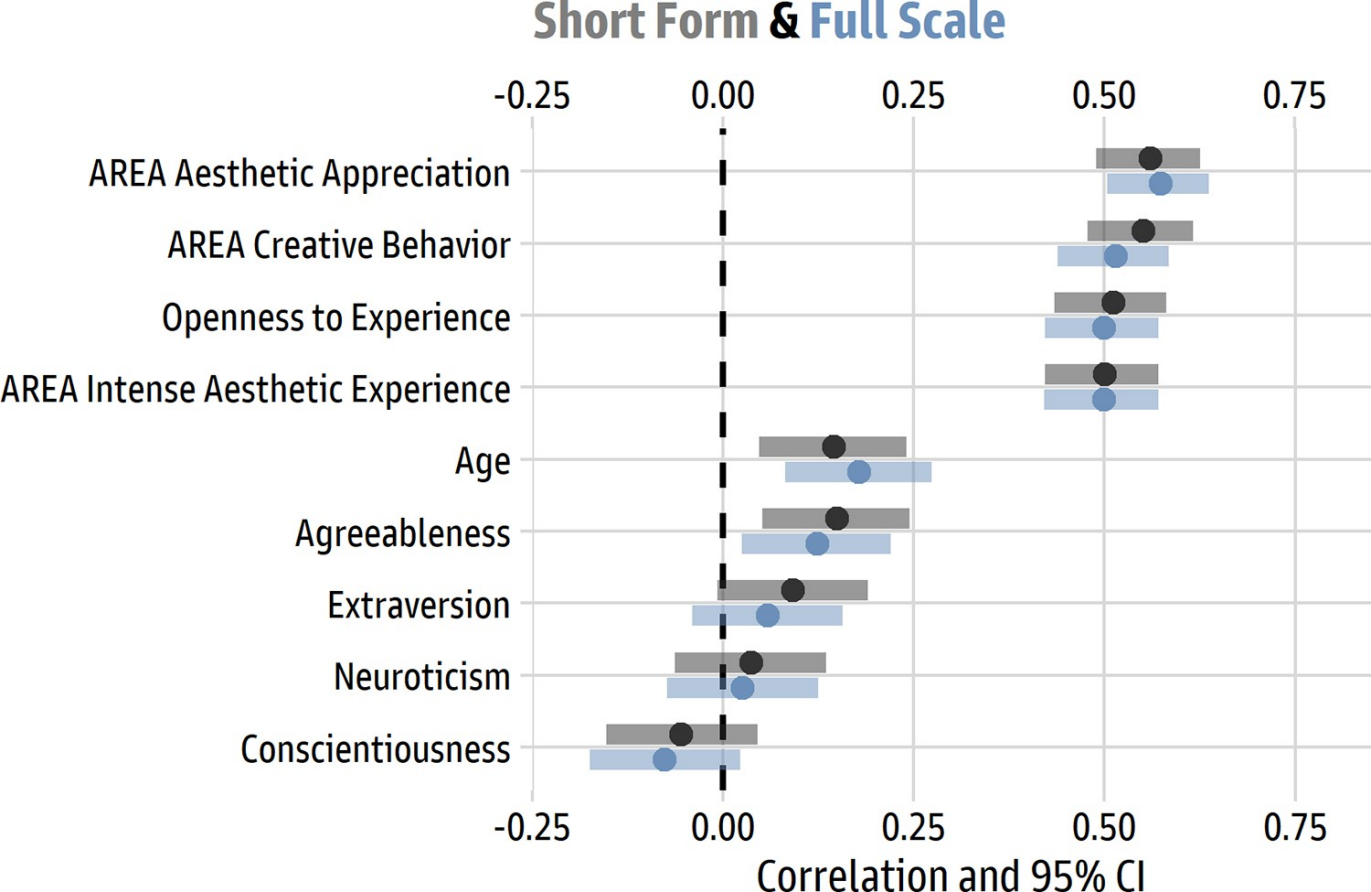
Pearson correlations for the short form and full scale. The dots represent the Pearson *r* correlation value; the bars represent the 95% confidence interval around *r*. The 10-item short form is illustrated in grey; the 26-item long form is illustrated in blue.

Using the data from Study 2, we explored the correlations between the short and long forms of the scale with personality traits, the AReA subscales, and age. As one would expect, the short form and full scale had essentially the same relationships with other variables (see Fig 10).

All told, the short form of the Revised Aesthetic Fluency Scale appears to reasonably represent the features of the long scale in light of the constraints for difficulty, coverage, and length. It is essentially unidimensional, has good internal consistency for a 10-item scale, and recovers the relationships found with the full scale. Short forms always represent a compromise, of course, but the short form of the Revised Aesthetic Fluency Scale appears promising for researchers who are working with constraints on participant time and survey length.

## Discussion

In the psychology of art, how much people know about art is fundamental to how they experience and encounter it. The original Aesthetic Fluency Scale [6] has been one of the most popular self-report tools for studying art knowledge, and the accumulation of research and practical experience over the years suggests some fruitful ways to improve the scale. The present research developed a Revised Aesthetic Fluency Scale that retains the same basic assessment approach as the original, but with some key changes.

First, the number of items was increased from 10 to 36, which allowed better coverage of the broad domain of Western art and to include a mix of important artists, art-historical concepts, and techniques and tools. As part of expanding the scale, the construct universe was sharpened to focus on Western fine art traditions in recognition of the impossibility of a relatively compact scale that does justice to the scope and richness of the world’s artistic traditions. Second, the response scale was refined by reducing the number of response options and refining the response labels. Third, the scales were developed to be “easier” so that their test information is higher for the ranges of art knowledge for which the scales are typically used. Finally, both longer and shorter forms of the scale were presented to meet the needs of different research contexts.

As a whole, the analyses indicate that the revised long and short scales are promising tools for arts researchers. Evidence for unidimensionality, internal-consistency reliability, local independence, orderly thresholds, and item fit was very good, and the scales’ difficulty level was appropriately lower so that it is better targeted for the typical populations that researchers study. There was no DIF based on past arts employment and minimal DIF based on gender. A single item in the long scale (“Frida Kahlo”) slightly favored women, but it was retained to balance DIF with representation of diverse artists. Initial evidence for score reliability came from sensible, expected differences in aesthetic fluency scores based on people’s interest in art and their past training, employment, and engagement with the arts (Study 1) and with well-known individual difference predictors of art engagement (Study 2).

For researchers interested in using the Revised Aesthetic Fluency Scale, we recommend the following best practices. First, the 36-item long form is the best choice in most experimental, laboratory, and survey research settings, whereas the 10-item short form is intended for when quicker assessments are required (e.g., museums and other field settings). Second, the intended population resembles that of the original scale: a wide range of knowledge, with an emphasis on art novices and the artistically engaged. Based on our test information curve, people with low knowledge (little experience with art; 2 *SD* below average) and very high knowledge (e.g., art experts; 4 *SD* above average) are measured relatively less reliably. As a result, this scale should not be used to define art expertise or to create extreme groups (i.e., selecting low scorers as “art novices” and high scorers as “art experts”) [10]. Finally, omega hierarchical (*ω_h_*), as assessed in this paper, is recommended for assessing internal consistency. Omega hierarchical disregards contributions of minor factors (e.g., correlated residuals) to capture the general factor [52], which is appropriate for the essentially unidimensional short and long form scales [34]. For convenience, researchers can use our Shiny app (https://alexander-christensen.shinyapps.io/rafs_app/) to compute a range of scores (e.g., sum scores and IRT factor scores) and internal consistency metrics (e.g., alpha and omega).

Regarding constraints on generality, our research recruited remote samples of adults residing in a handful of predominantly English speaking countries, and such a sampling strategy comes with well-known caveats concerning WEIRD samples [48] along with caveats concerning Internet access and willingness to join a paid research pool. In addition, while societal investment in art education is unfortunately uneven across the countries in our sample, in general adults’ interest in and engagement with the arts is inflected by complex issues of education, leisure, and social class [53]. Finally, we should emphasize that the Revised Aesthetic Fluency scale was designed with a broadly Western population in mind so that the universe of item content matched the common cultural and educational affordances of the participants’ culture. We do not see the scale as a useful tool for measuring general art knowledge in participants from non-Western cultures.

One of the influential aspects of the original Aesthetic Fluency Scale was its assessment approach: present important figures and terms from art history and ask people to rate how much they know about it. As noted earlier, some researchers have applied this approach to other domains (e.g., a film knowledge scale) [19] and to subscales of new instruments (e.g., the Art Affinity Index) [20]. We would encourage researchers to expand the portfolio of assessment tools for arts researchers by applying this versatile approach to new domains. In particular, we see a pressing need for assessment tools for domains outside of the traditional fine arts and for emerging art domains (e.g., street art). The need for tools for measuring knowledge of artistic traditions outside the Western canon is particularly acute. The Revised Aesthetic Fluency scale focuses on Western art, given the communities of researchers and populations who used the original scale, and tools devoted to studying other artistic traditions would be invaluable for the broader community of arts researchers.
